# Reduced Treatment-Emergent Sexual Dysfunction as a Potential Target in the Development of New Antidepressants

**DOI:** 10.1155/2013/256841

**Published:** 2013-02-04

**Authors:** David S. Baldwin, M. Carlotta Palazzo, Vasilios G. Masdrakis

**Affiliations:** ^1^Clinical and Experimental Sciences Academic Unit, Faculty of Medicine, University of Southampton, Southampton SO14 3DT, UK; ^2^Department of Psychiatry, University of Cape Town, Cape Town, South Africa; ^3^Department of Pathophysiology and Transplantation, University of Milan and Fondazione IRCCS Ca' Granda, Ospedale Maggiore Policlinico, 20122 Milan, Italy; ^4^First Department of Psychiatry, Eginition Hospital, Athens University Medical School, 11528 Athens, Greece

## Abstract

Pleasurable sexual activity is an essential component of many human relationships, providing a sense of physical, psychological, and social well-being. Epidemiological and clinical studies show that depressive symptoms and depressive illness are associated with impairments in sexual function and satisfaction, both in untreated and treated patients. The findings of randomized placebo-controlled trials demonstrate that most of the currently available antidepressant drugs are associated with the development or worsening of sexual dysfunction, in a substantial proportion of patients. Sexual difficulties during antidepressant treatment often resolve as depression lifts but can endure over long periods and may reduce self-esteem and affect mood and relationships adversely. Sexual dysfunction during antidepressant treatment is typically associated with many possible causes, but the risk and type of dysfunction vary with differing compounds and should be considered when making decisions about the relative merits and drawbacks of differing antidepressants. A range of interventions can be considered when managing patients with sexual dysfunction associated with antidepressants, including the prescription of phosphodiesterase-5 inhibitors, but none of these approaches can be considered “ideal.” As treatment-emergent sexual dysfunction is less frequent with certain drugs, presumably related to differences in their pharmacological properties, and because current management approaches are less than ideal, a reduced burden of treatment-emergent sexual dysfunction represents a tolerability target in the development of novel antidepressants.

## 1. Introduction

Systematic reviews of the epidemiology of sexual difficulties, dysfunction, and dissatisfaction indicate that sexual problems are common in men and women in all societies and more frequent in older individuals and among those with chronic medical conditions, including depression [1, 2]. For example, the Global Survey of Sexual Attitudes and Behavior, of over 27,000 men and women aged 40–80 years, found “early ejaculation” (i.e., rapid or premature ejaculation) to be the most common sexual dysfunction, affecting 14% of men, with “erectile difficulties” having a prevalence of 10% all sexual dysfunctions in men being more prevalent in older groups [3]. The Men's Attitudes to Life Events and Sexuality Study, of similar size but among men aged 20–75 years, found the prevalence of “erectile dysfunction” to be 16%, the proportion being higher in older men and individuals with cardiovascular disease, hypertension, or depression [4]. The Women's International Study of Health and Sexuality, in over 4,500 women aged 20–70 years, found “hypoactive sexual desire disorder” to have a prevalence range of 16–46%, in pre-menopausal to surgically postmenopausal women [5]. 

There is a close and two-way relationship between the presence of depressive symptoms and reports of sexual difficulties and dissatisfaction. Recognizing the nature and strength of this association, a recent international consensus statement on sexual dysfunction in patients with chronic illness recommends screening for depression [6]. The longitudinal epidemiological Zurich Study found the prevalence of sexual problems in depressed individuals (including those with major depression, dysthymia, and recurrent brief depression) to be approximately twice that in controls (50% *versus* 24%) [7]. Sexual problems may be more frequent in those with recurrent depression, as the United States Study of Women's Health Across the Nation found that only those with recurrent episodes were significantly more likely to report problems in sexual arousal, physical pleasure, and emotional satisfaction, when compared to controls [8]. 

Given its effects on mood, energy, capacity for pleasure, self-confidence, and self-esteem, it should be anticipated that depression would lower sexual interest and satisfaction; and this is the case, more markedly so in younger patients [9]. Depressive symptoms commonly coexist with anxiety symptoms, which are also associated with reports of sexual difficulties [10, 11] and often with obsessive-compulsive symptoms, known to be associated with loss of sexual pleasure and sexual dissatisfaction [12, 13]. But depression exerts adverse effects on the full range of the sexual response, including the ability to achieve and maintain penile erection or attain adequate vaginal moistening and to achieve ejaculation or orgasm [14]. Most antidepressant drugs can exert untoward effects on sexual function and satisfaction, but when considering the relative risks for and management of sexual dysfunction associated with antidepressant treatment, the adverse effects of depression itself—and of any coexisting physical illness or concomitant medication—can be easy to overlook.

## 2. Relative Incidence of ****Sexual Dysfunction during ****Antidepressant Treatment

Accurate identification of the incidence of “treatment-emergent” sexual dysfunction (including both the worsening of preexisting problems and the development of new sexual difficulties in previously untroubled patients) during antidepressant treatment has proved troublesome. Two international studies of the prevalence of sexual dysfunction in depressed patients prescribed either a selective serotonin reuptake inhibitor or serotonin-noradrenaline reuptake inhibitor, which take account of the presence of self-reported sexual problems prior to starting antidepressant and of the presence of concomitant medication sometimes implicated in causing sexual difficulties, suggest that 27–65% of female and 26–57% of male patients experience either a worsening of preexisting difficulties or the emergence of new sexual difficulties in the early stages of treatment, the differences in prevalence partly reflecting variations in case ascertainment and local clinical practice [15, 16]. 

Elucidation of the relative incidence of treatment-emergent sexual dysfunction with differing antidepressants has also proved difficult. Ideally, studies should be prospective, randomized, double-blind, and placebo-controlled in a defined diagnostic group, with an assessment of sexual function at baseline, and direct comparisons between drugs, prescribed at doses of equivalent efficacy. In addition, sexual dysfunction should be assessed with a reliable, valid and sensitive rating scale, rather than relying on reports or open questions which could be interpreted variably by different patients. Only a small proportion of investigations of treatment-emergent sexual dysfunction have met these rigorous criteria, but a series of meta-analyses together provide reasonable evidence that antidepressants differ in their propensity for worsening sexual function.

An early meta-analysis of studies with differing methodologies (including open-label, double-blind, cross-sectional, and retrospective investigations) indicates that “treatment-emergent sexual dysfunction” is no more common with agomelatine, amineptine, bupropion, moclobemide, mirtazapine, or nefazodone than it is with placebo, in contrast to the situation with other antidepressants [17] (Table 1): all other antidepressants were significantly more likely than placebo to be associated with sexual dysfunction, as a unitary category, and nearly all of these were significantly more likely to be associated with dysfunction in each stage of the normal sexual response. A meta-analysis of randomized controlled trials of the efficacy and tolerability of acute treatment of major depressive episodes with “second-generation” antidepressants indicates that bupropion is associated with a significantly lower rate of treatment-emergent sexual dysfunction than is seen with escitalopram, fluoxetine, paroxetine, or sertraline [18]: this is probably due to the nonserotonergic but predominantly noradrenergic-dopaminergic mechanism of action of bupropion [19]. A systematic review of the relative efficacy and tolerability of mirtazapine and comparator antidepressants in the acute treatment of major depression suggests that mirtazapine is significantly less likely than other antidepressants to cause adverse sexual effects [20], which is probably related to its antagonist effects at both the alpha-2 adrenergic receptor and the 5-HT_2C_ receptor [21]. Randomized controlled trials with agomelatine suggest it has fewer adverse effects on sexual functioning than some other antidepressants, which is more probably due to its antagonist effects on the 5-HT_2C_ receptor, rather than the agonist effects at melatonin receptors [22–24].

## 3. Assessing Sexual Functioning in ****Depressed Patients

There are many risk factors for developing sexual dysfunction during antidepressant treatment (including male gender, older age, lower academic achievement, lack of full-time employment, physical ill-health, multiple drug treatment, and troubled interpersonal relationships), but only some of these are amenable to intervention. Interindividual variations in pharmacokinetics may be important, as the “poor metabolizer” status for cytochrome P450 2D6 contributes to sexual dysfunction with paroxetine [25, 26], as does genetic variation in P-glycoprotein which affects its blood-brain barrier transfer [27]. Both patients and health professionals may find it embarrassing to mention and discuss sexual symptoms, consultation [28] and recognition rates in primary care are low [29], and recent reviews demonstrate that relying on the spontaneous reporting of adverse events can lead to a substantial underestimation of the prevalence of sexual problems [30, 31]. Comprehensive assessment of a depressed patient reporting sexual difficulties whilst undergoing antidepressant treatment can be a protracted process: although use of scales cannot fully substitute for a sensitive but comprehensive interview, the assessment can be facilitated by employing screening or severity questionnaires [32]. An early review suggested that few sexual functioning scales and questionnaires had sufficiently robust psychometric properties, but the Arizona Sexual Experiences Scale, the Changes in Sexual Functioning Questionnaire, the Psychotropic-Related Sexual Dysfunction Questionnaire, and the Sex Effects Scale all have adequate properties (including validity, reliability, and sensitivity to change) and so can be used to monitor sexual function and satisfaction prior to and during antidepressant treatment [32]. 

## 4. Improvement in Sexual Function during ****Antidepressant Treatment

Not all the sexual effects of antidepressants are unwanted in all patients. For example, many men troubled by persistent premature ejaculation can derive benefits from treatment with either clomipramine or selective serotonin reuptake inhibitors, on either a daily or “as required” basis [33], and a systematic review of randomized placebo-controlled trials with trazodone indicates that when prescribed at a higher dosage (150–200 mg per day), it is efficacious in reducing the “psychogenic” erectile dysfunction [34]. Whilst a substantial proportion of patients experience treatment-emergent sexual dysfunction whilst taking antidepressants [15, 16], the reduction of depressive symptoms through successful antidepressant treatment can also be accompanied by reported improvements in sexual desire and satisfaction [35, 36]. Whilst it is thought that treatment-emergent sexual dysfunction is a cause of nonadherence to antidepressants, the proportion of patients that stop treatment because of sexual problems is not established [37], and neither is the time-course of sexual dysfunction in patients who persist with antidepressant treatment [38].

## 5. Management of Sexual Dysfunction in Depression

The international studies demonstrate that the presence of sexual dysfunction associated with antidepressant treatment can significantly reduce self-esteem and quality of life, and also impose burdens on interpersonal relationships over and above those associated with depression [15, 16, 39]. Many approaches for managing patients troubled by sexual dysfunction associated with antidepressant drugs have been proposed, but the number of randomised placebo-controlled trials is limited, there is an absence of randomised controlled data evaluating the effect of psychological interventions [40], and none of the current approaches can be considered “ideal.”

If patients are concerned to preserve sexual functioning, choosing an antidepressant from the list of those regarded as having fewer adverse effects on sexual functioning (Table 1) is reasonable, when other considerations allow; however some of these antidepressants have other side effects, only limited availability, or questionable efficacy. Sexual side effects of at least some antidepressants may be dose-related, and a reduction in daily dosage is a commonly adopted first-line approach to management [41] (Table 2), but this approach may contribute to a relapse of symptoms and should be contemplated only for patients who have achieved a full symptomatic remission and who have successfully completed the continuation treatment. Regular brief interruptions of treatment (drug holidays) have previously been proposed as potentially useful for some antidepressants [42], but only a proportion of patients describe improvements in sexual function (and only with some antidepressants), depressive symptoms tend to worsen, and discontinuation symptoms can be troublesome [43], together making this approach to management potentially hazardous and consequently rather uncommon [41].

Many adjuvant compounds have been advocated for relieving sexual dysfunction associated with antidepressant drug treatment, though relatively few compounds have been subjected to rigorous evaluation. Randomised placebo-controlled trials indicate probable efficacy for bupropion [40], olanzapine [40], testosterone gel [44], and the phosphodiesterase-5 inhibitors sildenafil (both in male and female patients [45, 46]) and tadalafil [47]. Comparative studies are rare, though a placebo-controlled study found no evidence of efficacy for augmentation with mirtazapine, olanzapine, or yohimbine in female patients [48]. Augmentation of antidepressant treatment with aripiprazole is associated with improvements in sexual interest and satisfaction in female depressed patients, independent of the improvement in depressive symptoms [49]. Though switching from one antidepressant drug to another seems reasonable and is a commonly adopted practice [41]; placebo-controlled evidence of efficacy for this approach rests on a study of switching from sertraline to nefazodone [40]: furthermore switching away from one drug to another may lead to discontinuation symptoms, and the replacement drug may prove less effective in controlling depressive symptoms.

## 6. The Role of Phosphodiesterase-5 Inhibitors

Nitric oxide is involved in the physiology of the sexual response, in both men and women. In men, nitric oxide in the corpus cavernosum of the penis binds to guanylate cyclase receptors, which results in increased levels of cyclic guanosine monophosphate (cGMP), leading to smooth muscle relaxation (vasodilation) of the intimal cushions of the helicine arteries, which in turn leads to vasodilation, increased flow of blood into the spongy tissue of the penis, and resulting in erection. Sildenafil, tadalafil and vardenafil are potent and selective inhibitors of cGMP-specific phosphodiesterase type 5 (PDE5), which is responsible for degradation of cGMP in the corpus cavernosum. The molecular structure of sildenafil is similar to that of cGMP and acts as a competitive binding agent of PDE5 in the corpus cavernosum, resulting in more cGMP and facilitation of erection [50]. In women, the role of nitric oxide and its interplay with estrogen are less well understood, but phosphodiesterase type 5 inhibitor enhancement of nitric oxide-cGMP in nonadrenergic-noncholinergic signaling for women seems similar to men, and the release of nitric oxide results in vasodilatation in clitoral and vaginal tissues [51]. 

As noted above, a series of randomised placebo-controlled trials have demonstrated that PDE-5 inhibitors are efficacious in resolving sexual dysfunction associated with antidepressants [45–47]. In addition, studies of men with erectile dysfunction and depressive symptoms (but not undergoing antidepressant treatment) indicate that prescription of PDE-5 inhibitors is often accompanied by a reduction in depressive symptom severity, an enhancement of quality of life, and an improvement in interpersonal relationships [52, 53]. Furthermore, investigations in animal models indicate that nitric oxide activity is an important vulnerability factor in the Flinders rat depressive phenotype [54], that passage of PDE-5 inhibitors across the “blood-brain barrier” is possible [55], that sildenafil has antidepressant-like effects after central muscarinic receptor blockade [56], and that administration of sildenafil can lead to a reversal of reduced social interactive behavior [55]. Given these observations, it could be argued that the advent of treatment with PDE-5 inhibitors is a “game-changer” in the management of patients with sexual dysfunction associated with antidepressants. However like other potential treatment approaches, PDE-5 inhibitors are not “ideal,” having side effects such as headache, dyspepsia, and visual disturbances and needing to be used cautiously in patients with cardiovascular disease, which is a common comorbidity with depression.

## 7. Refined Approaches to Pharmacotherapy

It seems possible that a growing understanding of the influence of genetic polymorphisms may see the adoption of laboratory approaches to identifying patient subgroups at increased risk of developing sexual side effects of antidepressant treatment. The genome-wide association study associated with the STAR*D programme in the United States shows that ten single nucleotide polymorphisms (SNPs) may mediate the effects of bupropion on sexual side effects [57], and another genome-wide association study in Japan indicates that 11 SNPs are associated with sexual dysfunction associated with the antidepressants fluvoxamine, milnacipran, and paroxetine [58]. Smaller studies suggest that sexual dysfunction associated with selective serotonin reuptake inhibitors may be influenced by both the GG [59] and the AA [60] genotype of the 5-HT_2A_ receptor 1438 G/A polymorphism.

Future management options may be extended through the development of new antidepressant treatments with a lower risk of causing sexual problems. These could include compounds with effects on the 5-HT_1A_ receptor, or with noradrenaline reuptake inhibitor properties or even complementary approaches, such as the use of S-adenosyl-l-methionine (SAMe) [61], Maca root (Peruvian Ginseng) [62], or saffron [63]. At present, the evidence relating to the effects of drugs acting on the 5-HT_1A_ receptor is intriguing: the partial agonist buspirone has been used to reduce sexual dysfunction associated with selective serotonin reuptake inhibitors [64, 65], and the partial agonist gepirone improves sexual functioning in depressed men, independent of antidepressant or anxiolytic effects [66]. The novel antidepressant drug vilazodone, which has both selective serotonin reuptake inhibitor and 5-HT_1A_ partial agonist properties, appears to have a low incidence of adverse effects on sexual functioning [67, 68], as does the “multimodal” compound LuAA21004 (vortioxetine), whose pharmacological properties include full agonism at the 5-HT_1A_ receptor [69]. However, the experimental 5-HT_1A_ full agonist VML-670 was not efficacious in reversing sexual dysfunction associated with fluoxetine or paroxetine [70], and preclinical studies suggest that selective 5-HT_1A_ antagonists can both prevent and reverse fluoxetine-induced sexual dysfunction in rats [71]. 

Although the antidepressant efficacy of the selective noradrenaline reuptake inhibitor is limited [72], randomised controlled trials indicate that it probably has fewer adverse effects on sexual function than selective serotonin reuptake inhibitors [35, 73, 74]. These findings could encourage the further development of compounds with noradrenaline reuptake inhibitory properties as part of their mechanism of action: a proof-of-concept placebo-controlled study with the novel “triple reuptake inhibitor” amitifadine suggests that it has a low propensity to worsen sexual function in depressed patients [75], though the novel noradrenaline reuptake inhibitor LY22166884 was associated with significantly more sexual adverse events than placebo in a recent large placebo-controlled trial [7].

Whilst these findings suggest a prospect for novel antidepressant treatments, less likely to be associated with sexual adverse effects than many of the current medications, patient management currently rests on making the best use of the available treatments. This involves careful but sensitive enquiries to establish whether sexual difficulties are present, on choosing antidepressants with a lower likelihood of worsening sexual dysfunction, when other considerations allow; on judiciously reducing antidepressant dosage, when this is feasible; and on gaining greater familiarity with the potential benefits and drawbacks of phosphodiesterase-5 inhibitors and other adjuvant treatments. 

## Figures and Tables

**Table 1:** Estimated proportion and relative likelihood of treatment-emergent sexual dysfunction (derived from Serretti and Chiesa [17]).

Antidepressant	*n* (sample)	% sexual dysfunction	Odds ratio
Moclobemide	28	0.23	.22
Agomelatine	228	3.94	.25
Amineptine	29	7.14	.46
Nefazodone	50	0.46	.46
Bupropion	645	10.38	.75
**Placebo**	**605**	**14.2**	**1**
Mirtazapine	49	2.32	2.32
Fluvoxamine	244	25.81	3.27
Escitalopram	305	37.04	3.44
Duloxetine	274	41.60	4.36
Phenelzine	24	6.43	6.43
Imipramine	54	7.24	7.24
Fluoxetine	1718	70.76	15.59
Paroxetine	1261	16.86	16.36
Citalopram	654	78.59	20.27
Venlafaxine	610	79.83	24.42
Sertraline	970	80.3	27.43

**Table 2:** Commonly adopted strategies for managing sexual dysfunction associated with antidepressant drugs. Questionnaire survey, US psychiatrists, expertise in managing sexual dysfunction [41]. Percentages indicate the proportion of physicians using that strategy as their preferred intervention.

Dysfunction	Most frequently used treatment strategies first-, second-, and third-line interventions
Impaired libido—men and women	First. Adding a dopaminergic agent (37.9%) Second. Switching to another antidepressant (mostly bupropion) (44.8%) Third. Switching to another antidepressant (mostly bupropion) (31%)

Impaired arousal—women	First. Adding a dopaminergic agent (amantadine, bupropion, stimulants) (37.9%) Second. Adding a dopaminergic agent (amantadine, bupropion, stimulants) (20.4%) Third. Switching to another antidepressant (mostly bupropion) (34.5%)

Impaired arousal—men	First. Adding a dopaminergic agent (mostly stimulants) (31%) Second. Switching to another antidepressant (mostly bupropion) (31%) Third (a). Switching to another antidepressant (mostly bupropion) (37.9%) Third (b). Adding sildenafil, tadalafil, or vardenafil (mostly sildenafil or all three) (37.9%) (*a and b used by equal number of experts as a third choice*)

Impaired orgasm—women	First. Adding a dopaminergic agent (amantadine, stimulants) (34.5%) Second. Switching to another antidepressant (mostly bupropion) (31%) Third. Switching to another antidepressant (mostly bupropion) (27.5%)

Impaired orgasm —men	First (a). Adding a dopaminergic agent (stimulants) (31%) First (b). Decreasing the dose of antidepressant (31%) Second. Switching to another antidepressant (mostly bupropion) (34.5%) Third. Switching to another antidepressant (mostly bupropion) (31%) (*a and b used by equal number of experts as a first choice*)
